# New Theory of Tinea; Its Supposed Vegetable Origin

**Published:** 1842-09

**Authors:** 


*New Theory of Tinea; its supposed Vegetable Origin.*—A few years ago a young Hungarian physician, M. Gruby, announced, as the result of an extensive series of microscopical observations, that the cutaneous disease, known by the name of tinea or favus, is owing to the development and growth in the skin of cryptogamous plants of the genus fungus, which he has called micoderme. He tells us that they exhibit the aspect and form of such vegetable productions from their earliest appearance to their complete maturity, at which stage, if examined with a magnifying glass of two or three hundred powers, he has distinctly recognised the fruits or sporules in the interior of their cavities, as we observe to be the case in other fungi. He is of opinion that the disease is propagated by these seeds. This novel idea of the etiology of the disease has excited much attention among the French dermatologists; and it is but just to M. Gruby to acknowledge that the researches which have hitherto been made on the subject at the St. Louis Hospital in Paris, have tended to confirm the accuracy of his statements.

The treatment, naturally suggested by this theory of the disease, is that of applying caustics or whatever is calculated to destroy the vegetable substance.

M. Devergie has, during the last year, been giving an extensive trial to a solution of the acid nitrate of mercury (of the French codex): the spots become at first of a reddish yellow color, and subsequently black after the application.

M. Emery, of the same hospital, is in the habit of employing a strong solution of iodine for the same purpose, and it has appeared to answer extremely well.

In another article in the same journal, Dr. Petel recommends an epilatory ointment; as, in his own opinion, the only prospect of effecting a rapid cure depends upon extracting the hairs from the diseased spots. This gentleman's experience, however, cannot well be trusted to; as he tells us that tinea is never present where the hairs have fallen out, and that the method which nature follows is always by the removal of the hair. He tells us, too, that all internal and external remedies are of little or no use until the hairs are extracted, or fall out spontaneously. It would seem, from what he says, that the empirics, MM. Mahon, have more success in the cure of the disease than any of the regular practitioners in Paris: their plan consists in applying, first, a strong lixivial soap to the head, and then an epilatory plaster, which, when removed, draws the hairs off with it.

Dr. Pelet recommends the following formula for an epilatory ointment and powder:—

| | | |
|---|---|---|
| Take of | Soda (of commerce) - - | 60 parts. |
| | Slaked lime - - - - | 4 " |
| | Lard - - - - - | 120 " |
| | Mix together. | |
| Take of | Quicklime - - - - | 120 parts. |
| | Powdered Charcoal - - | 8 " |
| | Mix together. | |

After cutting the hair very short, the crusts are to be detached by applying linseed poultices to the scalp, and washing it repeatedly with soap and water. After several days' use of these means, all the affected parts are then to be rubbed with the ointment: this is to be repeated daily. The swelling and redness of the scalp gradually subside, but without ever ceasing entirely. The pustules, the successive reproduction of which keeps up the disease, become more rare and appear only at long intervals. To effect this change, from two to three or four months are often required. When this is the case, a pinch of the powder is to be sprinkled among the hair every second day or so. The hair becomes gradually loosened, so that it can be easily detached with a fingers or with a forceps, without causing pain. When the affected parts are rendered quite bald, the treatment is nearly at an end; all that is necessary is to anoint the head with a small portion of the ointment every two or three days, and to keep it exceedingly clean.—*N. Y. Lancet*, from *Bulletin de Therapeutique.*

